# Genome Sequencing of *Serinicoccus chungangensis* Strain CD08_5 Isolated from Duodenal Mucosa of a Celiac Disease Patient

**DOI:** 10.1128/genomeA.00043-16

**Published:** 2016-03-10

**Authors:** Atul Munish Chander, Gurwinder Kaur, Ramesan Girish Nair, Devinder Kumar Dhawan, Rakesh Kochhar, Shanmugam Mayilraj, Sanjay Kumar Bhadada

**Affiliations:** a Department of Biophysics, Panjab University, Chandigarh, India; b Microbial Type Culture Collection and Gene Bank (MTCC), CSIR-Institute of Microbial Technology, Chandigarh, India; c Department of Gastroenterology, Postgraduate Institute of Medical Education and Research, Chandigarh, India; d Department of Endocrinology, Postgraduate Institute of Medical Education and Research, Chandigarh, India

## Abstract

For the first time, we report here the 3.5-Mb genome of *Serinicoccus chungangensis* strain CD08_5, isolated from duodenal mucosa from a celiac disease (CD) patient. The specific annotations obtained revealed genes associated with virulence, disease, and defense, which predict its probable role in the pathogenesis of CD.

## GENOME ANNOUNCEMENT

Celiac disease (CD) is an autoimmune disorder of the small intestine, which causes intestinal inflammation resulting in progressive destruction of intestinal villi (villous atrophy), elongated crypts (crypt hyperplasia) (1), and altered intestinal barrier (2) in genetically predisposed subjects. Gluten (wheat protein) is considered the environmental factor responsible for disease pathogenesis. Human leukocyte antigen genes (HLA-DQ2/DQ8) are the genes associated with a risk of CD, but fewer than one-tenth of the carriers of these genes develop CD, suggesting the role of other genetic and environmental factors in the disease pathogenesis (1). In the recent emphasis on environmental factors, imbalanced gut microbiota is reported to be associated with CD (3–8). Looking at the emergence and reporting of unique intestinal microbes, whole-genome sequencing is very important for specific annotations that help predict their functional behavior and probable role in health and disease. Here, for the first time, we report the draft genome sequence of *Serinicoccus chungangensis* strain CD08_5, isolated from duodenal mucosa from a CD patient. The study was approved by the ethics committee of the Postgraduate Institute of Medical Research, Chandigarh, India, and a written consent was obtained from the participant.

Genomic DNA was extracted from 48-h-old culture using the ZR fungal/bacterial DNA MiniPrep kit, as per the manufacturer’s instructions. The genome of *S. chungangensis* strain CD08_5 was sequenced using the Illumina-HiSeq 1000 technology. A total of 13,847,293 reads were generated, amounting to 1,308,733,629 bp, and were *de novo* assembled using CLC Genomics Workbench version 7.5.1 (CLC bio, Aarhus, Denmark) into 34 contigs, with a total length of 3,556,982 bp and mean coverage of 100×. The assembly has an *N*_50_ of 297,860 bp, average contig length of 107,787 bp, and a mean G+C content of 72.9%. The functional annotation was carried out by Rapid Annotations using Subsystems Technology (RAST) (9), tRNAs were predicted by ARAGORN (10), and rRNA genes were predicted by RNAmmer 1.2 (11). The genome was predicted to contain a total of 3,160 coding sequences (CDSs), 3 rRNAs, and 53 tRNAs.

Whole-genome annotation available at the RAST server shows that *S. chungangensis* strain CD08_5 contains genes for vancomycin B-type resistance protein VanW, antibiotic efflux protein, copper resistance protein CopC, multidrug resistance transporter, Bcr/CflA family, arsenical resistance protein ACR3, β-lactamase class C and other penicillin-binding proteins, RNA-binding protein Jag, and VapC toxin protein. A functional comparison of the genome sequences available on the RAST server revealed the closest neighbor of *S. chungangensis* strain CD08_5 to be *Janibacter* sp. (score, 528), followed by *Kytococcus sedentarius* DSM 20547 (score, 432), *Renibacterium salmoninarum* ATCC 33209 (score, 276), and *Kineococcus radiotolerans* SRS 30216 (score, 269).

### Nucleotide sequence accession numbers.

This whole-genome shotgun project has been deposited at DDBJ/EMBL/GenBank under the accession number LQBL00000000. The version described in this paper is the first version, LQBL01000000.
